# Predictive power of a single body temperature at different cutoff values for neonates in the nursery transferring to special care nursery

**DOI:** 10.1097/MD.0000000000012619

**Published:** 2018-10-19

**Authors:** En-Pei Lee, Meng-Kung Yu, Shu-Chun Lee, Feng-Xia Gao, Han-Ping Wu

**Affiliations:** a Division of Pediatric General Medicine, Department of Pediatrics, Chang Gung Memorial Hospital at Linko, Kweishan; b College of Medicine, Chang Gung University; c Division of Pediatric Critical Care Medicine, Department of Pediatrics, Chang Gung Memorial Hospital at Linko, Kweishan; d Department of Pediatric Emergency Medicine, Children's Hospital, China Medical University; e Department of Pediatrics; f Department of Nursing, Taichung Tzuchi Hospital, the Buddhist Medical Foundation; g Asia University; h Department of Medical Research, Children's Hospital; i Department of Medicine, School of Medicine, China Medical University, Taichung, Taiwan.

**Keywords:** body temperature, neonatal hyperthermia, newborn, nursery, special care nursery

## Abstract

The aim of this study was to identify the clinical parameters indicative of serious etiology of neonatal hyperthermia and to determine the appropriate cutoff value of body temperature (BT) for predicting the need to transfer the newborn to the special care (SC) nursery.

The nursery records of newborns diagnosed with hyperthermia between 2007 and 2013 were retrospectively reviewed. The clinical characteristics of newborns with hyperthermia remained in the nursery were compared with those transferred to the SC nursery. In addition, the receiver operating characteristic analysis was used to determine the appropriate cutoff BT for predicting further septic workup in the SC nursery.

Among the 92 newborns with hyperthermia evaluated, 30 (32.6%) were transferred to the SC nursery and 62 (67.4%) remained in the nursery. Clinical characteristics associated with transfer to the SC nursery included the highest BT, BT at first measurement during hyperthermia, frequency of hyperthermia, duration of hyperthermia, irritable crying, decreased appetite, poor activity, vomiting with abdominal distension, tachypnea, and tachycardia (all *P* < .05). BT for predicting the need for transferring newborns with hyperthermia to the SC nursery had an area under the curve of 0.976 (*P* < .001). A BT of 38 °C was determined as the optimal cutoff value for predicting the need to monitoring for suspicious clinical symptoms (sensitivity (Sn), 93%; specificity (Sp), 87%). Furthermore, BT≥38.2 °C (Sn, 70%; Sp 100%) and BT≤37.8 °C (Sn, 100%; Sp, 61%) respectively were determined as the cutoff values for transferring newborns to the SC nursery or allowing them to remain in the regular nursery.

Our results suggest a BT of 38 °C represents the optimal cutoff indicating newborns for close monitoring for suspicious clinical presentations including irritable crying, decreased appetite, poor activity, vomiting with abdominal distension, tachypnea, and tachycardia. Newborns with BT < 37.8 °C may remain in the nursery but should be transferred to the SC nursery for septic workup and empiric antibiotics if the BT is above 38.2 °C.

## 1 Introduction

Hyperthermia is defined as a core temperature greater than 37.3°C to 38.3°C, without a change in the body's temperature set point.^[1–4]^ In contrast, fever occurs when the body's temperature set point is higher. The difference between fever and hyperthermia is the underlying mechanism. Specifically, hyperthermia represents elevated body temperature (BT) because of failed thermoregulation that occurs when a body produces or absorbs more heat than it dissipates, whereas fever may have infectious and non-infectious etiology. Therefore, determining whether a newborn has hyperthermia or fever is a challenging issue in the clinical setting.

Neonatal hyperthermia is considered an alarming condition indicative of hidden systemic infections. It is important that pediatricians and nurses can rapidly identify newborns with hyperthermia that require transfer for further investigation and treatment. In infants aged up to 90 days, assessing rectal temperature is the standard method for detecting fever, which is defined as a rectal temperature of 38°C or greater.^[1–3]^ Nevertheless, evaluating newborns with fever and deciding whether they should be transferred to the special care (SC) nursery or remain in the regular nursery is sternly challenging in clinical practice. In recent decades, increasing awareness of this issue has led to efforts to discriminate which newborns with fever might need more versus less intensive management. The commonly used criteria such as the Rochester, Philadelphia, and Boston protocol were designed for identifying febrile infants with serious bacterial infection.^[4,5,7]^ Although measuring BT represents an objective and non-invasive approach to evaluate whether interventions such as further septic workup should be considered or not,^[6,8–11]^ it is not entirely clear what BT cutoff values are optimal for predicting serious etiology of hyperthermia in babies during the first 3 days of life. In addition, the set of clinical symptoms and signs indicating serious etiology of neonatal hyperthermia have not been described. Therefore in a population of term well-being babies without risk factors for infection admitted to the nursery after birth, we aimed to describe the clinical parameters indicating serious etiology of hyperthermia in newborns and to determine the optimal BT cutoff value for predicting neonatal sepsis and the need for further septic workup and empiric antibiotics in the SC nursery.

## 2 Materials and methods

### 2.1 Study population

A retrospective review of nursery charts was performed for newborns diagnosed with hyperthermia between January 2007 and December 2013 at a single center. The following inclusion criteria were applied: well-being term babies without risk factors for infection after birth, gestational age, >37 weeks; body weight at birth, >2500 g; and diagnosis of neonatal hyperthermia. The following exclusion criteria were applied: history of prematurity, birth trauma, preinatal asphyxia, major congenital malformations, maternal premature rupture of membrane for over 18 hours, prenatal infection, and maternal infection such as chorioamnionitis or group B streptococcus infection with antibiotics usage less than 4 hours. The study was approved by the Human Subjects Review Committee of Asia University in Taiwan

### 2.2 Data collection and analysis

The following variables were recorded and analyzed: sex, gestational age, body weight at birth, vital signs, delivery method (normal spontaneous delivery or cesarean section), rooming-in allowance and duration, feeding method (exclusively breast fed or mixed feeding), body weight loss, and activity of the infant. According to the World Health Organization guidelines, exclusive breast feeding is the optimal feeding method for newborns. However, when the amount of breast milk was insufficient, the newborns also received formula, and the feeding method was classified as mixed feeding. Initially all included newborns were checked their BTs by using the axillary thermometry, but once the temperature was higher than 37.3 °C, the neonates were rechecked and confirmed their BT by rectal thermometry.^[12–15]^ Readings were obtained using a digital thermometer (Digiclassic MT101 Hardtip Thermometer; China). When a newborn in the nursery was identified as having hyperthermia, the BT was measured by rectal thermometry about every 30 minutes until the neonate's temperature was within normal range then resumed to every 4 hours until discharge. The newborn will stay in the nursery for first 3 days of life in the hospital. Key information regarding these measurements was also recorded, including the highest BT, BT at the first measurement during hyperthermia, frequency of hyperthermia, duration of hyperthermia, and severity of dehydration. For newborns referred to the SC nursery, the results of laboratory tests and imaging examinations, as well as the clinical course of hospitalization were also recorded.

For the purpose of the present analysis, newborns with hyperthermia were divided into 2 groups. Newborns that remained in the nursery for observation were included in the nursery group. While those who were transferred to the SC nursery for further surveillance were included in the SC nursery group if they had hyperthermia more than 39 °C or hyperthermia less than 39 °C and had at least one of the following presentations: irritability, feeding difficulties, poor activity, abdominal distention, apnea, desaturation, tachycardia, or bradycardia.^[16,17]^ Clinical characteristics were compared between the 2 groups to identify the relevant risk factors for the need for being transferred to the SC nursery. Subsequently, a receiver operating characteristic (ROC) analysis was performed to determine the appropriate BT cutoff value for evaluating the necessity of monitoring a newborn with hyperthermia for suspicious clinical presentations. Finally, 2 BT cutoff values were determined that can more easily be applied to clinical practice in deciding when a newborn with hyperthermia should be transferred to the SC nursery or may remain in the regular nursery.

### 2.3 Statistical analysis

Statistical analyses were performed using the *t* test, Mann–Whitney *U* test, chi-square test, and Fisher exact test, as well as the ROC curve analysis. The results of the descriptive analyses of independent variables were reported as percentages and mean ± standard deviation (SD). Differences between the 2 groups were presented with their 95% confidence intervals. The significance threshold was set at 0.05. Predictive power for each BT cutoff value was described in terms of the sensitivity, specificity, area under the ROC curve, positive likelihood ratio (LR^+^), and negative likelihood ratio (LR^–^). The area under the ROC curve, calculated using the trapezoidal rule, was considered as a global measure of the diagnostic value for a given BT cutoff. LR^+^ and LR^–^ were calculated only for the best performing cutoff values. The criterion value was the BT cutoff with the highest accuracy (minimal percentage of false-negative and false-positive results). Statistical analyses were performed using SPSS version 15.0 (SPSS Inc., Chicago, IL).

## 3 Results

Of the 2152 newborns with a gestational age of over 37 weeks and a body weight at birth above 2500 g registered during the study period, 92 newborns with hyperthermia were included in our study. Among them, 30 newborns (32.6%) were transferred to the SC nursery for further surveillance and management, while 62 (67.4%) remained under observation in the regular nursery. In addition, 41 newborns (44.6%) were male while 51 (55.4%) were female, with a mean gestational age at birth of 39 ± 1.2 weeks (Table 1). The frequency of hyperthermia was 2.6 ± 0.7, and the duration of hyperthermia was 3.9 ± 4.3 hours. The highest BT was 37.9 ± 0.35 °C and the first BT recorded during hyperthermia was 37.8 ± 0.33 °C. Of the 92 newborns included in the study, 87 (94.6%) had a full 24-hour rooming-in period and 74 (80.4%) were exclusively breast fed. The age of first hyperthermia was 37.1 ± 15.7 hours.

**Table 1:**
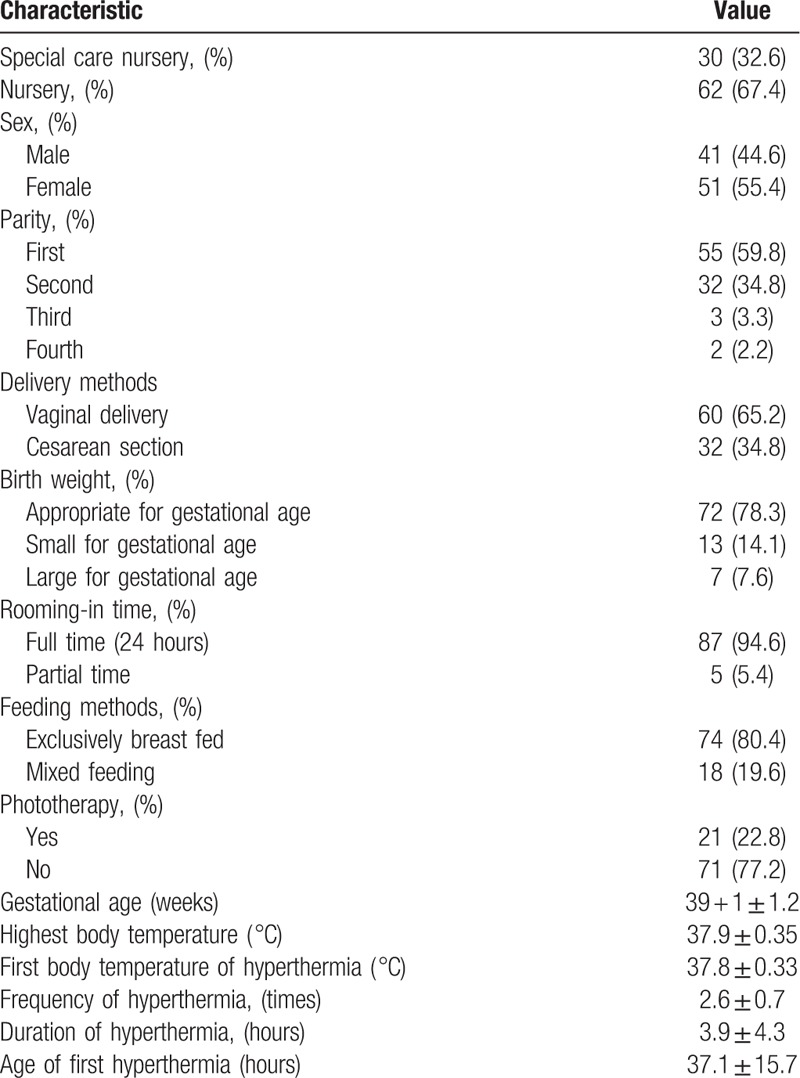
Demographics of 92 newborns with hyperthermia.

The clinical characteristics of newborns with hyperthermia were compared between the nursery and SC nursery groups, and the results are shown in Table 2. The clinical characteristics that differed significantly between the 2 groups included irritable crying, decrease of appetite, vomiting with abdominal fullness, decreased activity, tachypnea, and tachycardia (all *P* < .05).

**Table 2:**
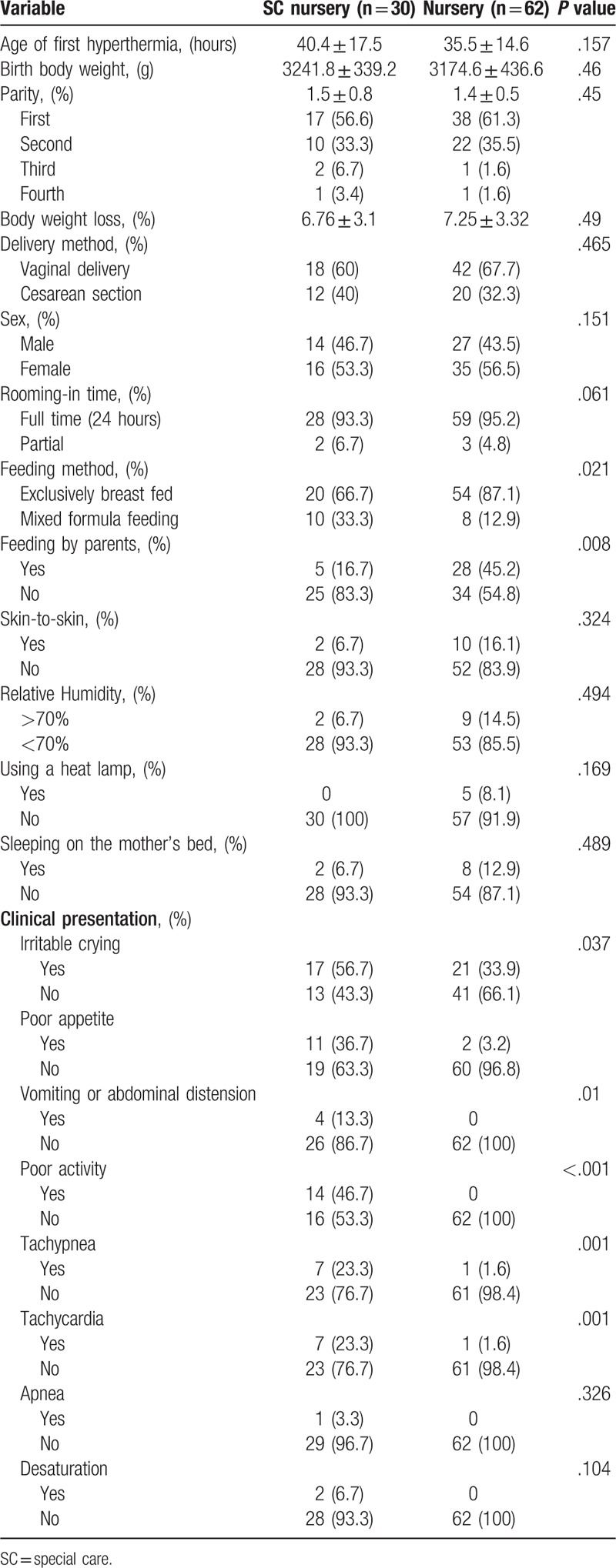
Comparison of demographic and clinical characteristics between newborns with hyperthermia who remained in the nursery and those who were transferred to the SC nursery.

Table 3 showed the discharge diagnosis of newborns who were transferred to the SC nursery which urinary tract infection was the major disease (12 newborns, 40%), followed by pneumonia (7 newborns, 23.3%) and bacteremia (5 newborns, 16.7%). In all the discharge diagnosis, culture-proven etiologies accounted for 18 (60%) and the other diagnosis were made based on criteria.^[18,19]^

**Table 3:**
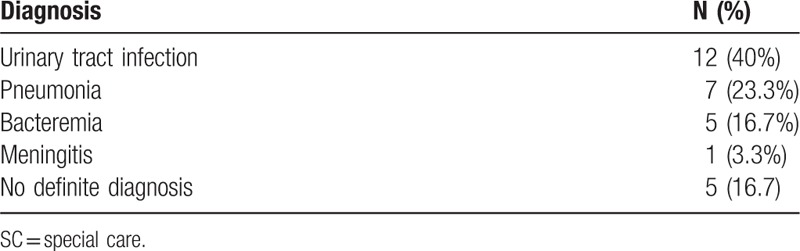
Discharge diagnosis of newborns who were transferred to the SC nursery.

**Figure 1:**
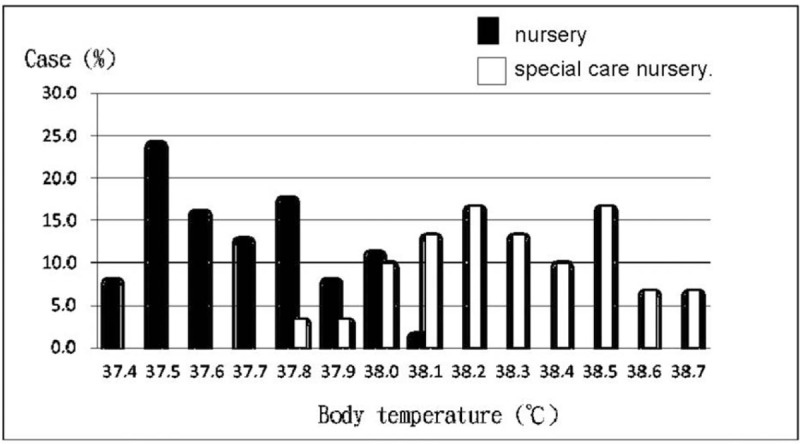
The distribution of body temperatures in newborns with hyperthermia who remained in the nursery and those who were transferred to the SC nursery. SC = special care.

In terms of BT (Table 4), the highest BT was significantly higher in the SC nursery group than in the nursery group (38.3 ± 0.23 °C versus 37.7 ± 0.19 °C, *P* < .001). In addition, the BT at the first measurement for hyperthermia, the frequency of hyperthermia, and the duration of hyperthermia were all significantly higher in the SC nursery group than in the nursery group (all *P* < .05). The distribution of BT values (Fig. 1) indicated that all BTs were below 38.2 °C in the nursery group, whereas all BTs were above 37.7 °C in the SC nursery group.

**Table 4:**
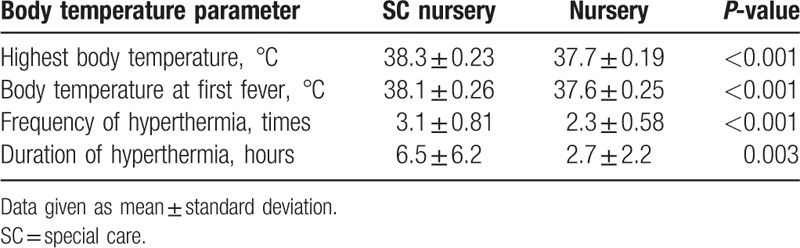
Comparative analysis of body temperature parameters in newborns with hyperthermia who remained in the nursery and those who were transferred to the SC nursery.

**Figure 2:**
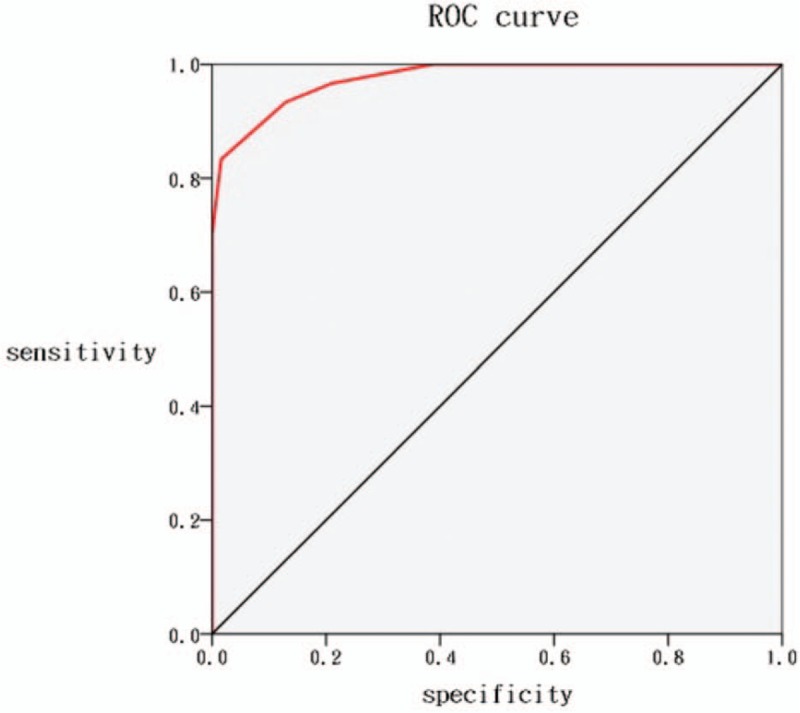
Receiver operating characteristic curves for body temperature predicting whether the newborn may remain in the nursery or should be transferred to the special care nursery.

Based on the results of the ROC curve analysis (Fig. 2), we determined the appropriate BT cutoff value for predicting the indication for transfer to the SC nursery or for remaining in the nursery. The area under the ROC curve was 0.976 (95% confidence interval, 0.95 to 1.00, *P* < .001). Table 5 provides an overview of the predictive accuracy (sensitivity, specificity, LR^+^, LR^–^, and Youden's index) for various BT cutoffs potentially relevant for the decision to transfer a newborn to the SC nursery. Among newborns with BT≤37.8 °C, none need to be transferred to the SC nursery for further evaluations (sensitivity, 100%; specificity, 61.0%; LR^+^, 2.58). On the other hand, newborns with BT>38.2 °C should be transferred to the SC nursery for further evaluations (sensitivity 70.0%; specificity 100%; LR^–^, 0.3). However, regarding newborns with the BT between 37.8 °C and 38.2 °C, close monitoring of clinical manifestations is necessary. Moreover, the results indicate that 38 °C is the optimal BT cutoff for predicting potentially neonatal sepsis and the newborn should be transferred to the SC nursery for septic workup and empiric antibiotics (sensitivity, 93%; specificity, 87%; LR^+^, 7.23; LR^–^, 0.08).

**Table 5:**
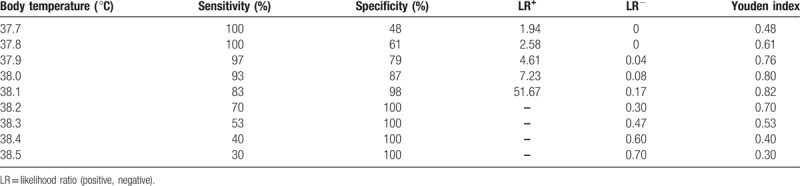
Body temperature cutoff values for predicting the need to transfer newborns with hyperthermia to the special care nursery for further septic workup.

## 4 Discussion

BT is 1 of the key parameters for continuous monitoring of the health status of infants in the nursery, SC nursery, and neonatal intensive care unit,^[20–22]^ as BT serves as an indicator for impeding infections and physiologic stability of the infant. Hyperthermia without symptoms/signs is common in healthy-term newborns in the nursery. Newborns with hyperthermia should be carefully evaluated to assess the underlying cause, which may be temperature dysregulation or insidious infection.^[10,11]^ How to wisely and safely investigate and manage the newborn with hyperthermia is a much-debated issue in the clinical setting. The present analysis of nursery records of newborns with hyperthermia identified several clinical characteristics to be considered when making a decision regarding whether the newborn should remain under observation in the nursery or be transferred to the SC nursery. The risk factors for transferring to the SC nursery included higher peak BT, higher BT at the first measurement for hyperthermia, longer duration of hyperthermia, and increased frequency of hyperthermia noted in the nursery. Furthermore, analysis of the discriminating capacity of BT cutoffs found that the BT may serve as an effective indicator in distinguishing sick newborns from healthy newborns. Specifically, a BT cutoff value of 38.0 °C was useful in predicting potentially severe etiology of neonatal hyperthermia and may provide a straightforward indicator for identifying newborns with hyperthermia that should be closely monitored either in the nursery or in the SC nursery.

Overall, from a clinical perspective, the analyses for evaluating neonatal infections in newborns with hyperthermia can be divided into 3 zones, 1 with high sensitivity, 1 with high specificity, and an indeterminate zone. The present study proposed 2 BT cutoffs that are more easily applicable in the clinical setting to ascertain at what point the threat of a severe underlying condition can be ruled in or ruled out in newborns with hyperthermia. Based on these results, BT was highly indicative of a severe condition when the newborn had a BT of 38.2 °C or above, and therefore such newborns should be immediately transferred to the SC nursery for further investigation. However, the newborns with hyperthermia may remain for observation in the nursery if the BT is 37.8 °C or lower. In this situation, there may not be a need for further investigation. Nevertheless, primary clinicians should carefully evaluate newborns with the BT in the indeterminate zone (i.e., between 37.8 °C and 38.2 °C) by assessing more clinical characteristics and obtaining further information to aid in the decision to keep the newborns with hyperthermia in the nursery or to transfer them to the SC nursery. Based on the present results regarding the clinical characteristics associated with transfer to the SC nursery, newborns with hyperthermia and BT in the indeterminate zone (between 37.8 °C and 38.2 °C) should be transferred to the SC nursery for further surveillance and management once they present with irritable crying, decreased appetite, poor activity, vomiting with abdominal distension, tachypnea and tachycardia. However, if the newborns are exclusively breast fed and fed by parents feeding but exhibit none of the above-described suspicious symptoms and signs, they may remain in the nursery for clinical observation.

Accurate and immediate diagnosis of neonatal early-onset sepsis is difficult because the signs and symptoms may be subtle and nonspecific initially. Therefore it's important to have a high index of suspicion when a newborn deviates the usual pattern of daily life. Previous studies have demonstrated that temperature instability especially persistent hyperthermia and higher temperature are highly indicative of infection which accounts for more than 50% of cases.^[23–25]^ Cardiopulmonary symptoms such as tachycardia and tachypnea are also common symptoms in infected neonates (≧50%of cases) but apnea is less common and is more likely in preterm infants or in late-onset sepsis.^[23,26]^ Other symptoms such as poor feeding (25 to 50% of cases), irritability and abdominal distension (10– 25% of cases) are also the meaningful risk factors for neonatal sepsis.^[23,26]^

Nevertheless, there were some limitations in this study. First, given geographical and country differences in clinical practice, the difference of patient selection may exist. Second, we analyzed a relatively small number of newborns in the study. In addition, the retrospective study was conducted only in 1 hospital, and there may be a potential risk of information bias. Therefore, future studies conducting in multi-centers with larger population are warranted.

## 5 Conclusions

Overall, the results of the present study suggest that 38 °C is a suitable cutoff for BT to indicate newborns for close monitoring of suspicious clinical presentations including irritable crying, decreased appetite, poor activity, vomiting with abdominal distension, tachypnea, and tachycardia. In addition, the results suggest that newborns with hyperthermia may remain under observation in the nursery if their BT is below 37.8 °C, but they should be transferred to the SC nursery for further septic workup and management if their BT is above 38.2 °C.

## Author contributions

HPW and MKY reviewed the medical records, analyzed and interpreted the data, and drafted the manuscript; EPL, FXG and SCL analyzed and interpreted the data. HPW designed and oversaw the study, interpreted the data, and revised the manuscript. All authors have read and approved the final manuscript for publication.

**Data curation:** Shu-Chun Lee.

**Investigation:** Shu-Chun Lee, Feng-Xia Gao, Han-Ping Wu.

**Methodology:** Han-Ping Wu.

**Supervision:** Han-Ping Wu.

**Writing – original draft:** En-Pei Lee, Meng-Kung Yu.

**Writing – review & editing:** Han-Ping Wu.
